# Leader behaviors to improve teamwork during interprofessional rounds in the NICU

**DOI:** 10.1038/s41372-026-02689-8

**Published:** 2026-04-21

**Authors:** Allison M. Traylor, Elizabeth A. Bell, Noelani C. M. Lewis, Gabrielle A. Rufrano, Bryan L. Ohning

**Affiliations:** 1 Clemson University, Clemson, SC USA; 2 Independent consultant, Atlanta, Georgia; 3 University of South Carolina School of Medicine–Greenville, Greenville, SC USA

**Keywords:** Health occupations, Paediatrics

## Abstract

**Objective:**

We investigated the impact of leaders’ interprofessional teamwork behaviors on NICU staff perceptions of teamwork processes, trust, psychological safety, and voice behavior.

**Study design:**

To develop a measure of leaders’ interprofessional teamwork behaviors, we observed 199 rounding events at a NICU in the Southeastern United States. We evaluated the effect of leaders’ teamwork behaviors on teamwork by observing 931 interprofessional rounds and collecting 115 staff surveys.

**Results:**

We identified three categories of leaders’ interprofessional teamwork behaviors during rounds: sharing information, inviting input, and maintaining positive rapport. Results indicated a small, positive effect on staff ratings of psychological safety and voice behavior. Leader behaviors did not significantly predict staff ratings of teamwork processes or trust.

**Conclusion:**

Results indicate that NICU leaders’ interprofessional teamwork behaviors can improve NICU staff perceptions of psychological safety and voice behavior during rounds, but did not indicate that these behaviors significantly affect teamwork processes or trust.

## Leader behaviors to improve teamwork during interprofessional rounds in the NICU

Effective teamwork is essential in the neonatal intensive care unit (NICU), where patient acuity is high and the context is complex and dynamic [1, 2]. Across studies, effective teamwork is associated with reduced medical errors [3], infection rates [2], and length of stay [4] in the NICU. Further, effective teamwork is associated with positive outcomes for staff including reduced moral distress amongst NICU nurses [5]. As a result, there has been increased interest in how to improve teamwork in the NICU. For example, several studies have found that team training can improve teamwork in the NICU [1, 6, 7]. Other work has identified critical moments wherein teamwork is particularly consequential, such as during neonatal resuscitation [6, 8, 9].

Interprofessional rounding provides another, less explored, context during which effective interactions may improve staff perceptions of teamwork [10]. Interprofessional rounding brings together clinicians and staff from different disciplines to share information and collaboratively arrive at a plan of care [11]. Across medical contexts, interventions to improve teamwork during rounding have had positive impacts on an array of patient and care quality/delivery outcomes [12, 13]. However, most of the studies in this area have focused on comparing approaches for structuring the information shared during rounds rather than on interpersonal behaviors that can support building teamwork. Although it is important to understand differences in structured rounding approaches, the interpersonal interactions among NICU staff (i.e., physicians, nurse practitioners, nurses, respiratory therapists, etc.) during rounds may influence perceptions of teamwork in the NICU. For example, prior work has emphasized the important role leaders play to build psychological safety in the NICU [14]; however, it is less clear how leaders can build psychological safety during interprofessional bedside rounds.

The purpose of this study was to identify the physician and neonatal nurse practitioner (NNP) behaviors that are positively associated with staff members’ perceptions of teamwork during interprofessional bedside rounds. Toward this effort, we (a) developed a tool to measure physician and NNP teamwork behaviors during rounds in the NICU and (b) tested the effect of these behaviors on staff perceptions of teamwork processes, psychological safety, voice, and trust. We focused on physicians and neonatal nurse practitioners, because these individuals often lead interprofessional bedside rounding in the NICU have an outsized impact on many teamwork outcomes [14]. We hypothesized that when physicians and NNPs displayed effective interprofessional teamwork behaviors during rounding, NICU staff would report higher levels of teamwork processes, psychological safety, voice, and trust.

## Methods

This study was approved by the Clemson University Institutional Review Board under protocol IRB2022–0691. Survey participants provided oral consent prior to participating in the study.

### Research context

We conducted this research in a NICU at a regional perinatal center in the southeastern United States. This NICU contained two units: an intensive care unit and an intermediate care unit. Leaders and the structure of rounds are consistent across these two units. Nine attending neonatologists were observed as part of this research study. Attending neonatologists lead pediatric residents and/or neonatal nurse practitioners (NNPs) to conduct interprofessional rounds. Each day, physicians and/or NNPs round on 20–25 patients across both the intensive and intermediate units. Rounds take place at the bedside over the course of two to four hours. Rounds typically take place with a patient’s nurse present. In addition, rounds frequently include a nutritionist, pharmacist, respiratory therapist, family support specialist, nursing or medical students, consulting physicians, other medical professionals, and patient family members.

### Behavioral scale development

To develop our behavioral measure of leader behaviors during patient rounding, the lead author conducted observations over 10 days, yielding 20 h of observation time and *N* = 199 observed patient rounding events. The lead author is an organizational psychologist whose primary area of expertise is team science. These observations focused on how team leaders (i.e., attending physicians, NNPs, and resident physicians) interacted with nurses, respiratory therapists, pharmacists, nutritionists, and other staff during interprofessional bedside rounds. The researcher observed leader behaviors for seven attending physicians and a rotating team of resident physicians and NNPs.

During observations, the researcher recorded detailed notes focused on how the physicians and NNPs interacted with NICU staff. No identifiable information was retained from these observations. The researcher made efforts to observe unobtrusively to avoid impacting how the team worked together. NICU staff were not aware of the purpose of the researcher’s observations, other than that the research was focused broadly on teamwork. The last author on this manuscript was a physician in this NICU, but was excluded from observations due to prior knowledge of our hypotheses. Between rounding events, the researcher occasionally asked clarifying questions or sought additional information about the NICU context from attending physicians, resident physicians, and NNPs.

We used qualitative content analysis to analyze the observational data to generate broader themes to summarize relevant leader behaviors [15]. Qualitative content analysis is an approach that may include both inductive and deductive methods. Given that we were pursuing a relatively nascent area of research, we used a conventional content analysis approach to analyze our qualitative data. Conventional content analysis is an inductive coding approach appropriate for research exploring a nascent area or describing a phenomenon. Authors using conventional content analysis are instructed to avoid using pre-existing frameworks to inform analysis.

First, we reviewed all observation notes, focusing on interactions between physicians or NNPs and other NICU medical team members. Next, we used an open coding approach to generate first-order codes from observations using NVivo qualitative analysis software. First order codes captured themes that were “close to the data.” For example, the first order code, “sought input from respiratory therapist” was used when a physician asked a respiratory therapist for their opinion on plans to change a patient’s ventilator settings. From there, the author generated second-order thematic codes to combine related first-order codes, organizing the data into three broad themes that could be used as the basis for a behavioral measure. Next, to develop the behavioral measure, the researcher developed a rating sheet with the three themes characterizing leader interactions with staff during rounds: sharing information, inviting input, and maintaining positive rapport. Table 1 provides an overview of the themes and descriptions of each category that were provided to raters.

**Table 1:** Leader behaviors during NICU rounding.

Leader Behavior Dimension	Description	Example
Sharing Information	Communicates changes and care with NICU staff; shares information from consults/external sources; educates where relevant; uses closed-loop communication	A neonatologist shares changes in the infant’s care plan with the team members
Inviting Input	Ensures relevant staff members present for rounds; solicits staff member expertise in decision-making; responds to questions without judgment; signals openness to input nonverbally	A neonatologist asks an RN for their expert opinion on decisions regarding the infant’s care plan
Maintaining Positive Rapport	Displays empathy or care to NICU staff members when appropriate; uses humor or jokes when appropriate; checks in on how staff members are doing; offers backup to staff members	A resident asks an RT how they are doing during the round

To establish content validity of our qualitative data and behavioral measures, subject matter experts including physicians and other NICU personnel reviewed our themes and behavioral measures prior to moving forward with the study. The research team made changes to the wording of items and descriptions of each sub-scale based on this feedback. Further, the qualitative findings and behavioral measures were reviewed by researchers with expertise in team science to ensure the content of these measures accurately captured teamwork constructs. We made additional revisions based on this feedback from team scientists. Finally, the research team conducted a series of pilot observations to ensure that the wording of observational tool subscales was clear.

### Procedure

To collect data for our main study, teams of two researchers attended daily patient rounding in the NICU. One researcher focused on observing and rating leader behaviors during rounds. The second researcher focused on collecting survey data from NICU nurses and other staff members (e.g., respiratory therapists, nutritionists, or pharmacists) who were present for rounding. Prior to collecting observational data, each researcher was trained on how to conduct observations using the behavior rating scale. Four researchers were trained to collect observational data. To train researchers and establish inter-rater reliability, two researchers from our team were assigned to observe each rounding event for 14 days (*N* = 257 rounding events). During this time, researchers discussed inconsistencies to come to agreement about the most accurate rating for each rounding event. The inter-rater reliability for the behavioral scale during our preliminary observations was excellent [16] (*ICC*(1,1) = 0.87), so we moved forward with a single observer. Preliminary observational data was excluded from further analyses.

We conducted observations during patient rounds between May and August 2023 (*N* = 931 patient rounding events). During observations, researchers recorded the staff members present for rounds (e.g., nurses, patient family members, etc.) and observed interactions between team leaders (i.e., physicians and NNPs) and staff who were present for rounding. Observers made notes on behaviors that related to the three behavioral dimensions that were the focus of this research: sharing information, inviting input, and maintaining positive rapport. At the conclusion of each rounding event, observers determined a numerical rating for each dimension on a scale ranging from *1 (rare/never)* to *5 (frequently)*. No identifying information was collected during observations. To ensure consistency in observations over time, observers were encouraged to discuss rounding events with the researcher who was collecting survey data during rounds. Further, data collection occurred over less than three months, which helped to avoid changes in ratings over time.

Survey data was collected following each observed rounding event. After each round concluded and physicians and NNPs had moved to another bedside, a researcher approached staff who were present for rounding to invite them to take part in our study. Staff members were instructed to complete a brief survey focused on their perceptions of teamwork with the physicians and/or NNPs with whom they had most recently rounded. Surveys contained measures of teamwork perceptions, psychological safety, voice, and trust. Researchers collected completed surveys from staff members within one hour of distributing the surveys. To ensure data from observations and surveys could be matched, researchers assigned a number to each rounding event and wrote the number on top of surveys before they were distributed to NICU staff; however, researchers did not collect any identifying information from staff members.

### Measures

The measures used in this study were collected from two sources. Leader teamwork behaviors were collected from researcher observations of interprofessional rounds. Leader teamwork behaviors were measured on a five-point Likert-type rating scale. Teamwork perceptions, psychological safety, voice, and trust were assessed via self-report survey measures completed by NICU staff after each observed rounding event (e.g., nurses or respiratory therapists). Survey items were also measured on a five-point Likert-type scale. We report the calculated Cronbach’s alpha for each scale. Cronbach’s alpha is a measure of internal consistency that is used as an indicator of reliability for psychometric scales.

### Leader teamwork behaviors

Ratings of leader teamwork behaviors were made by trained observers. The leader teamwork behaviors scale captured three behavioral dimensions (Cronbach’s *α* = .70): sharing information, inviting input, and maintaining positive rapport. Examples of these three dimensions are included in Table 1.

### Teamwork perceptions

Staff surveys included 10 items [17] to measure teamwork behavior (Cronbach’s *α* = .93), which were adapted slightly to reflect the medical context. An example item included, “To what extent does this team actively work to ensure that everyone on our team clearly understands our goals regarding patient care?”

### Psychological safety

Staff surveys included two items [18] to measure psychological safety (Cronbach’s *α* = .53). Items included, “If I made a mistake on this team, it would be held against me,” and “It is difficult to ask members of this team for help.” Both items were reverse scored.

### Voice

Staff surveys included two items [19] to measure voice behavior (Cronbach’s *α* = .75). Items included, “I proactively give suggestions for issues that may influence this team,” and “I proactively voice constructive suggestions which are beneficial to this team.”

### Trust

Staff surveys included two items [20] to measure trust (Cronbach’s *α* = 0.82). Items included, “I am able to count on this team for help if I have difficulties with my job,” and “I trust these team members.”

### Sample

Our sample contains survey data from 115 NICU staff. Our sample size was selected to achieve adequate power based on an expected small effect (*f* ^2^ = 0.10). Survey data was matched with observations for each of these rounds. 92.9% of the surveys collected were completed by nurses and 7.1% were completed by respiratory therapists or other NICU staff members. Nurses were present for 90.6% of observed rounding events. Respiratory therapists were present for 16.1% of observed rounding events. Notably, respiratory therapists were present for 32.5% of rounds for patients in the critical care unit, and only 0.6% of rounds for patients who were not in the critical care unit due to a low incidence of respiratory support in this patient population. Other medical team members (e.g., pharmacist, nutritionist, etc.) were present during 62.5% of observed rounds. Patient family members were present during 13.0% of rounding events. To protect the confidentiality of survey respondents, we did not collect demographic information outside of the respondent’s professional role. Further, we did not collect information on the professional roles of team members other than nurses and respiratory therapists to protect the identity of these participants as the unit employs such a small number of pharmacists and nutritionists.

### Statistical analysis

All statistical analyses were conducted using R statistical software. Our data for this study were multilevel, as researchers observed one physician/NNP team each day, collecting both observational ratings and surveys from daily rounding events with the same leaders. As a result, the dataset included “rounding events” (Level 1) nested within physician/NNP teams (Level 2). We calculated *ICC*(1) values to estimate the reliability of group membership. *ICC*(1) values can be interpreted as the percentage of the variance in individual perceptions of a variable that can be “explained” by group membership [21]. For example, the *ICC*(1) value for psychological safety by NICU teams was *ICC*(1) = 0.022, indicating that 2.2% of variability in participants’ perceptions of psychological safety could be attributed to team membership. We found that *ICC*(1) values were not statistically significant for teamwork processes (*ICC*(1) < 0.001, *p* = 0.97), psychological safety, (*ICC*(1) = 0.022, *p* = 0.24), voice behavior (*ICC*(1) = 0.060, *p* = 0.45), nor trust (*ICC*(1) = 0.001, *p* = 0.85). In addition, there was no significant variability in the regression slope of observations and outcome variables between teams. That is, there was not sufficient between-team variability to justify using multilevel modeling to analyze the data [21]. We also tested *ICC*(1) values for each of our outcomes to assess whether the NICU where rounds took place (i.e., intermediate vs. intensive) or the individual attending physician leading the rounds explained significant variability in our outcome variables. Our results indicated that physician leader and NICU location predicted less than 2% of variability in any of our outcome variables, so we proceeded with a single-level model.

Accordingly, we used simple linear regression to test the effect of leader behaviors on our four outcome variables. In our results, we report *b*, or the unstandardized beta weight for the relationship between leader teamwork behaviors and each of our outcomes. Accordingly, *b* can be interpreted as the change in ratings for each outcome variable for every one scale point increase in leader teamwork behaviors. All study measures were assessed on a five-point Likert-type scale. The *p*-values presented are for two-tailed statistical tests.

## Results

Descriptive statistics and correlations between variables are included in Table 2. Notably, staff ratings of both team behavioral processes (*M* = 4.25, *SD* = 0.51) and trust (*M* = 4.40, *SD* = 0.52) were close to the upper end of our five-point scale. These results suggest a “ceiling effect” that the vast majority of participants “agreed” or “strongly agreed” with our items for team behavioral processes and trust.

**Table 2:** Means, standard deviations, and correlations between study variables.

Variable	*M*	*SD*	1	2	3	4
1. Leader Teamwork Behaviors^a^	2.49	0.86				
2. Team Behavioral Processes^b^	4.25	0.51	0.12			
3. Psychological Safety^b^	4.06	0.67	0.20*	0.32**		
4. Voice^b^	3.99	0.58	0.19*	0.28**	0.23*	
5. Trust^b^	4.40	0.52	0.02	0.66**	0.38**	0.20*

*M* and *SD* are used to represent mean and standard deviation for each study variable. The values contained in off-diagonal cells represent the correlation between two variables (e.g., the correlation between leader teamwork behaviors and team behavioral processes is 0.12). All variables were measured on a 5-point scale with higher scores indicating more favorable ratings. * indicates *p* < 0.05. ** indicates *p* < 0.01.

^a^Observer ratings.

^b^Staff survey ratings.

### Team behavioral processes

First, we explored the relationship between leader teamwork behaviors and staff members’ perceptions of team behavioral processes. Leader teamwork behaviors did not predict staff ratings of team behavioral processes, *b* = 0.07, *t*(113) = 1.32, *p* = 0.19. Further, leader teamwork behaviors explained only 0.15% of variability in team behavioral processes (*R*^*2*^ = 0.02, *F*(1, 113) = 1.74, *p* = 0.19).

### Psychological safety

Next, we explored the relationship between leader teamwork behaviors and staff members’ perceptions of psychological safety. Leader teamwork behaviors predicted psychological safety, *b* = 0.16, *t*(113) = 2.21, *p* = 0.029. Leader teamwork behaviors explained 4.15% of variability in psychological safety (*R*^*2*^ = 0.04), *F*(1, 113) = 4.89, *p* = 0.029.

### Voice behavior

Next, we explored the relationship between leader teamwork behaviors and staff members’ reported voice behavior. Leader teamwork behaviors predicted staff voice behavior, *b* = 0.13, *t*(113) = 2.03, *p* = 0.045. Leader teamwork behaviors explained 3.52% of variability in voice behavior (*R*^*2*^ = 0.04), *F*(1, 113) = 4.13, *p* = 0.045.

### Trust

Finally, we explored the relationship between leader teamwork behaviors and staff members’ perceptions of trust. Leader teamwork behaviors did not predict staff members’ perceptions of trust, *b* = .01, *t*(113) = 0.18, *p* = 0.86. Further, leader teamwork behaviors explained only 0.02% of variability in team trust (*R*^*2*^ < 0.01), *F*(1, 113) = 0.03, *p* = 0.86.

## Discussion

Our results provide evidence that NICU leaders’ (i.e., physicians and NNPs) teamwork behaviors during rounds were positively related to staff members’ perceptions of psychological safety and reported voice behavior during interprofessional rounds. Although our results indicated a positive relationship trend between NICU leaders’ teamwork behaviors and staff perceptions of teamwork processes and team trust, these relationships were not statistically significant. Taken together, these findings indicate that leaders’ teamwork behaviors, including sharing information, inviting input, and maintaining positive rapport during rounding events are related to beliefs about one’s ability to speak up (i.e., psychological safety), as well as whether staff members voice their concerns during rounds (i.e., voice behavior). Improving psychological safety climate and increasing voice behavior is essential in the NICU and across healthcare contexts, where speaking up about medical errors or other concerns can have life or death consequences [22–24]. In addition, psychological safety is an important predictor of team learning [25, 26]. Given that educating staff and medical trainees is often a focus of interprofessional bedside rounds, it is important to understand how to enhance team learning in this context [27].

These findings also expand upon growing literature suggesting that leader behaviors during interprofessional medical rounds can positively influence teamwork and other important staff outcomes. However, while previous studies have explored the effect of structured rounding on team outcomes, structured rounding protocols generally focus on whether task-relevant information is shared during rounds [12]. Structured rounding and other approaches that codify the information communicated between nurses and physicians during rounds yield more effective teamwork and satisfaction, including in intensive care settings [13, 28]. However, structured rounding protocols often focus strictly on what information must be shared during rounds via a checklist or list of questions [12].

While our observational data captured some of the same elements that are typically ascribed in structured rounds (e.g., ensuring all team members are present, sharing relevant information), our study suggests that team leaders display other important behaviors during rounds that may not be included in a structured rounding protocol (Table 1). Indeed, structured rounding was in place in the NICU that served as the context for these studies. However, even with structured rounding in place, there was some variability in whether and how the structure was implemented. Further, there was more substantial variability in how physician/NNP leaders engaged in behaviors that are not typically prescribed by a structured rounding protocol. For example, positive responses to nurses’ questions or input and checking in on staff members’ well-being were emphasized in our behavioral scale but are not typically considered in structured rounding protocols [12].

To improve psychological safety and voice behavior during rounding, individuals designing structured rounding protocols might consider incorporating the leader behaviors captured in our study. For example, the “sharing information” dimension could be incorporated into structured rounding by including a checklist of information that should be shared during rounds, including information from consults or other external sources. The “inviting input” dimension could be incorporated by including a section in a structured rounding protocol for actively soliciting input or expertise from each medical team member at the bedside during rounds. Finally, the “maintaining positive rapport” dimension could be structured by including a very brief “check in” on to assess how team members are doing and whether any staff members need help or backup.

The findings from our study also have implications for increasing “speaking up” during interprofessional bedside rounding as well as designing interventions to improve leadership in the NICU. First, guidelines for patient rounding may be expanded to include recommendations for nonverbal communication. For example, responding openly to others’ suggestions is a behavior highlighted in our study that is strongly related to psychological safety [29]. Future research may investigate how emphasizing this behavior in structured rounding or training leaders on responding openly during rounds could improve outcomes. Second, “speaking up” interventions in healthcare, which focus on improving psychological safety and voice, are often implemented through staff training initiatives [30]. Our findings suggest that approaches to improve leader behavior during rounds may be an alternative way to improve speaking up behavior in medical contexts. Finally, the behavioral rating tool developed for our study could be incorporated in quality improvement initiatives or leader coaching to improve leader behaviors. For example, quality improvement initiatives could include observational ratings of these leader behaviors as a pre- and post- measure to assess implementation of a structured rounding protocol. A coach could use the tool to conduct observations of NICU leaders and use the associated notes and ratings to provide feedback on leader behavior during rounding.

Counter to our expectations, we did not find support for a relationship between individual leader teamwork behaviors and staff perceptions of teamwork processes or trust. There are two reasons why this may be the case. First, mean scores for both teamwork processes and trust were quite high (Table 2). As a result, there may have been a ceiling effect, such that effective leader behavior could not increase perceptions of teamwork behaviors or team trust measurably higher. Second, it is possible that behavior during a single rounding event is insufficient for improving perceptions of teamwork processes or trust. Indeed, interventions to improve psychological safety and voice often focus on improving brief interactions, such as communication during rounds [31]. In contrast, interventions to improve teamwork processes, such as team training, are often more time intensive and focused on a wider range of behaviors that occur outside of rounding [32]. As a result, it may not be possible to significantly improve perceptions of team behavioral processes or trust in a single rounding event.

Our study design had several notable strengths. First, our primary analysis used data from multiple sources including observational data collected using a behavioral rating scale and survey data from nurses, respiratory therapists, and other staff members. Accordingly, our study design should assuage concerns about common method variance, which are common in behavioral research [33]. Second, our primary analysis used observational data to predict outcomes. The structured observation protocol used in this research should address concerns about subjectivity during observational data collection. Finally, we used qualitative data from observations of interprofessional rounds to design our behavioral rating scale. This qualitative data improves the richness of our findings as we were able to provide observers with more specific information about the type of leader behaviors encountered during interprofessional rounding.

As is the case with any study, this work also has some notable limitations. First, our study design did not allow for random assignment. Future research could incorporate the behaviors identified here in a structured rounding protocol and implement a protocol using a randomized control trial to overcome this limitation.

In addition, there may be limitations to the generalizability of our findings. Our study took place in a single NICU. Although we expect these findings would generalize to other NICU environments, future research may consider leader rounding behavior in other hospitals. In addition, our study considered interactions between NICU leaders and staff only during interprofessional rounding. As a result, we could not capture the full scope of interactions that may impact perceptions of teamwork outcomes.

Our inability to collect data from other sites and relatively brief staff surveys limits our ability to establish the validity of our behavioral scale. Accordingly, future research might test this tool in other NICU contexts and collect additional measures of leader behavior as further evidence of content validity and convergent or discriminant validity, respectively. Further, unmeasured confounds such as workload, census, or burnout may have affected psychological safety independent of leader teamwork behavior. Future studies may measure these variables to account for potential confounding effects. Relatedly, our two-item measure of psychological safety exhibited low reliability (α = .53). Low reliability suggests that the two items selected for our survey were not highly correlated. One potential reason for this may be related to participant responses to the item, “It is difficult to ask members of this team for help.” Participants in the study were primarily nurses and respiratory therapists who were asked to respond to items about the leading physician and NNP team. In this NICU, it is unlikely that nurses would ask physicians for help completing their daily tasks. As a result, this item may not have been the best indicator of psychological safety with leader teams. Future studies should include additional items to measure psychological safety [25] to address this issue.

Finally, as identified earlier in our discussion, our study may have been limited by a ceiling effect for staff ratings of trust and teamwork perceptions. Although it is good that staff had such high ratings of trust and teamwork in our study, extremely high scores on these dimensions may have limited our ability to detect differences in teamwork processes or trust that resulted from leader behavior during rounds.

## Conclusion

Our findings indicate that effective leader behavior during rounds (i.e., sharing information, inviting input, and maintaining positive rapport) improves psychological safety climate and voice behavior amongst medical staff in the NICU. Although structured rounding interventions are often focused on improving information sharing and inviting input, our findings suggest that even when structured rounding is in place, there is substantial variability in leader behavior during interprofessional rounds. Thus, we suggest that future research explore how expanding structured rounding approaches to include nonverbal behavior to invite input (e.g., positive responses to staff input) and to maintain positive rapport with staff members (e.g., checking in on staff member well-being or using appropriate humor during rounds) might further improve teamwork.

## Data Availability

The datasets generated during and analyzed in the current study are available from the corresponding author on reasonable request.
