# Correction: Leader behaviors to improve teamwork during interprofessional rounds in the NICU

**DOI:** 10.1038/s41372-026-02713-x

**Published:** 2026-05-26

**Authors:** Allison M. Traylor, Elizabeth A. Bell, Noelani C. M. Lewis, Gabrielle A. Rufrano, Bryan L. Ohning

**Affiliations:** 1 Clemson University, Clemson, SC USA; 2 Independent consultant, Atlanta, Georgia; 3 University of South Carolina School of Medicine–Greenville, Greenville, SC USA

**Keywords:** Health occupations, Paediatrics

Correction to: *Journal of Perinatology* https://doi.org/10.1038/s41372-026-02689-8, published online 21 April 2026

In the original version of this article, the Competing Interest Statement and an Author Note have been typeset as a single section in the article. This correction now has split these two sections into their respective parts.

The original publication has been corrected.

